# Evolving Concepts: Immunity in Oncology from Targets to Treatments

**DOI:** 10.1155/2015/847383

**Published:** 2015-04-28

**Authors:** Hina Khan, Rasim Gucalp, Iuliana Shapira

**Affiliations:** ^1^Department of Hematology Oncology, Montefiore Medical Center, Albert Einstein School of Medicine, 1300 Morris Park Avenue, Bronx, NY 10461, USA; ^2^Department of Oncology, Montefiore Medical Center, Albert Einstein School of Medicine, 1300 Morris Park Avenue, Bronx, NY 10461, USA; ^3^Department of Hematology Oncology, Hofstra North Shore LIJ School of Medicine, Hempstead, NY 11549, USA

## Abstract

Cancer is associated with global immune suppression of the host. Malignancy-induced immune suppressive effect can be circumvented by blocking the immune checkpoint and tip the immune balance in favor of immune stimulation and unleash cytotoxic effects on cancer cells. Human antibodies directed against immune checkpoint proteins: cytotoxic T lymphocytes antigen-4 (CTLA-4) and programmed death-1 (PD-1), programmed death-ligand 1 (PD-L1), have shown therapeutic efficacy in advanced melanoma and non-small-cell lung cancer and other malignancies. Immune check point blockade antibodies lead to diminished tolerance to self and enhanced immune ability to recognize and eliminate cancer cells. As a class these agents have immune-related adverse events due to decreased ability of effector immune cells to discriminate between self and non-self. Seventy percent of patients participating in clinical trials have experienced anticancer activities and varying degrees of immune mediated dose-limiting side effects.

## 1. Background

*Global Immune Suppression Precedes the Development of Overt Malignancy: Mechanisms*. Developing malignancies evade immune detection due to failure of T lymphocytes to recognize and respond to tumor specific antigens. Patients with advanced metastatic disease and large tumor burdens manifest a global immune suppression as evidenced by decrease response to challenge with common antigens and diminished T-cell function [1–5].

Epidemiological studies suggest occult malignancy is associated with global immune suppression and diminished immune surveillance with manifestations such as zoster, tuberculosis, and viral reactivation. Clinical reactivation of herpes zoster precedes overt cancer by more than 800 days [6–8].

T-cells are able to recognize and eliminate foreign antigens when presented to T-cell receptor (TCR) in the context of self-major histocompatibility complex (MHC), to activate immune responses following TCR binding a second, more adhesive, signal to create immune synapse necessary for T-cell activation. The second signal comes from cell-cell interaction between CD28 on T-cell and B-7 receptors (either CD80 or CD86) on antigen presenting cells (APC). Once T-cell is activated it becomes CD8+, effector T-cell capable of recognizing and eliminating cells marked by foreign antigen and generating intracellular signals producing interleukin-2 (IL-2) a cytokine that promotes T-cell proliferation [9].

Activated T-cells start expressing CTLA-4 receptor, an immune check point receptor, which has a higher binding affinity for B-7 ligand than CD28. CTLA4 displaces CD28 from B7 receptors leading to termination of effector immune responses and establishment of tolerance for the antigen presented minimizing the danger of autoimmunity. In activated T-cells CTLA4 is induced by ligation of CD28 to B7 ligands while T-regulatory cells (CD4+ CD25+) constitutively express CTLA4.

In addition interactions between B-7 on APC and immune check point receptors PD-1 and or CTLA4 lead to production of arginase and indolamine dioxygenase (IDO) [9]. IDO decreases T-cell access to tryptophan starvation, transforms tryptophan to N formyl-kynurenine inducing T-cell apoptosis [10] further decreasing cytotoxic effector T-cell responses to tumor associated antigens (TAA). High IDO expression is an independent prognostic variable for reduced overall survival in cancer patients [11]. Arginase depletes tumor microenvironment from the essential amino acid arginine needed for zeta chain synthesis of the T-cell [12], the principal signal-transduction element of the T-cell receptor (TCR), without arginine T-cells becoming anergic [13, 14]. Arginase and indolamine dioxygenase deplete the microenvironment from arginine and tryptophan, two essential amino acids critical for CD8+ T-cell survival; absence of these amino acids leads to CD8+ cell anergy and death [15].

Programmed death-1 (PD-1) is a protein that belongs to CD-28 family and is expressed on T-cells, dendritic cells, natural killer cells, macrophages, and B-cells [16]. PD-1 is not expressed on resting T-cells but is inducible, appearing within 24 h after stimulation and T-cell activation [17] (Table 1).

PD-1 has three known ligands PD-L1 (programmed death ligand-1), PD-L2 (programmed death ligand-2), and B7-1 (CD80) [18]. PD-L1 can be expressed by T-cells, B-cells, myeloid dendritic cells, and at very low levels tissue macrophages in the lung, kidney, liver, heart, and placenta [19, 20]. PD-L1 is constitutively expressed on many solid and hematological malignancies [21–30] (Table 1). When PD-1 binds to PD-L1 and PD-L2 (programmed death ligand), the immune responses are dampened and T-cell becomes unresponsive [31]. PD-L1 binding to B7-1 on antigen reactive T-cells inhibits late stage T-cell responses [18] and limits the response to inflammation [30, 32–34]. Blockade of PD-1 on regulatory T-cells (CD4+, CD25+) inhibits their ability to mediate tolerance [35–37] (Table 1).

**Figure 2:**
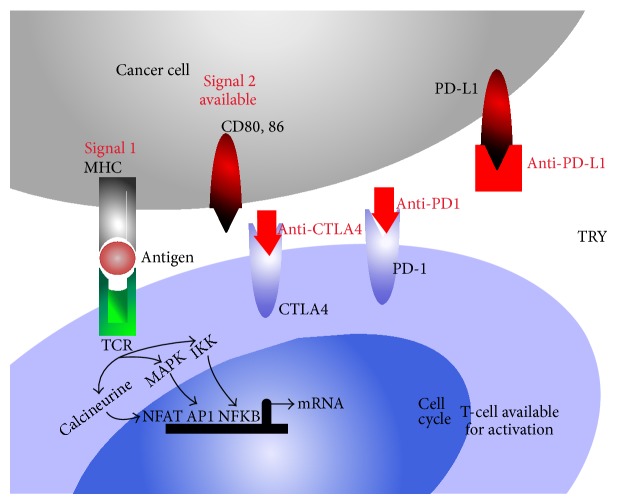
Immune therapy in cancer: blocking CTLA4-B7 interactions to enhance T-cell activation could help overcome tumor antigen tolerance and consequently potentiate enhanced antitumoral responses. Blocking PD-1, PD-L1 allows for more B7 family members on immune cells such as CD80/CD86 to be available and bind CD28 (Figure 1) allowing for more activated effector T-cells capable to recognize and eliminate cells bearing cancer antigens. TCR: T-cell receptor; MHC: major histocompatibility antigen; IL-2: interleukin-2; CTLA4: cytotoxic T lymphocytes antigen-4; PD-1: programmed death-1 also known as CD279; PD-L1: programmed death-ligand 1 known as B7-H1 or CD274; PD-L2: programmed death-ligand 2, known as CD273 or B7-DC B-7 dendritic cell; IFN-*γ*: interferon gamma; IL-2: interleukin-2; APC: antigen presenting cells; TRY: tryptophan; AP-1: activator protein-1; NFAT: nuclear factor of activated T-cells; NFKB: nuclear factor kappa B; MAPK: mitogen activated protein kinase; IKK: I kappa B kinase; PI3K: phosphatidylinositol-4,5-biphosphate 3-kinase; CDK: cyclin dependant kinase; JAK3: Janus kinase 3; mTOR: mammalian target of rapamycin; anti-CTLA4: antibody blocking CTLA4; anti-PD-1: antibody blocking PD-1; anti-PD-L1: antibody blocking PD-L1.

Blocking humanized monoclonal antibodies against cytotoxic T lymphocyte antigen-4-mediated PD-1, PD-L1, and PD-L2 prevents binding to (CD80/CD86) allowing them to be available for CD28 binding and T-cell activation and decreasing immune tolerance to tumor associated antigens [38, 39] (Figure 2).

Malignant cells express members of B7 family of receptors such as CD80, CD86, PD-L1, PD-L2, and ICOS (Table 1), with high affinity for CTLA4 and or PD-1 converting T-cells into anergic and tolerant regulatory T-cells (T-regs).

In various cancer types high expression of PD-L1 on tumor cells and to a lesser extent of PD-L2 has been found to correlate with poor prognosis and survival [40].

Levels of circulating immune suppressor cells are three to five times higher in patients with advanced head and neck, non-small-cell lung cancer, pancreatic cancer, and colon and breast cancer, when compared to normal controls [41] supporting the conclusion that immune anergy, tolerance, and suppression are central not only to cancer development but also to cancer progression [42].

The initial research attempts to manipulating immune response by stimulating effector T-cells in antigen-independent fashion were done using a humanized CD-28 molecule TGN1412. TGN1412 activated CD28 positive effector T-cells with antitumor activity in animal models of cancer including in primates. In a phase I clinical trial TGN1412 induced a “cytokine storm” in all six enrolled participants leading to life-threatening multiorgan failure in normal human volunteers. Current immune check point inhibitors anti-CTLA-4, anti-PD-1, and anti-PDL1 therapies are not antigen specific thus able to bind common molecular epitopes that are expressed on the targeted as well as on the nontargeted T-cells [43].

## 2. Immune Check Point Inhibitors in Use and Clinical Trials

*(1) Ipilimumab.* A fully humanized IgG1 monoclonal antibody [BMS-734016] recognizing CTLA-4 interferes with CTLA-B7 interactions on the surface of antigen presenting cells, permitting CD28-B7 complex formation. Since 2011, Ipilimumab is approved for the treatment of unresectable or metastatic melanoma at 3 mg/kg intravenous every 3 weeks for a total of 4 doses. It is currently undergoing trials for the treatment of non-small-cell lung carcinoma, bladder cancer, and metastatic castrate resistant prostate cancer (Table 2).

*(2) Tremelimumab.* A CTLA-4 blocking Ig G2 monoclonal antibody showed durable responses in advanced melanoma patients in early phase studies; however in phase III trial at dose of 15 mg/kg versus standard chemotherapy, Tremelimumab showed no survival benefits. It is being investigated in colorectal, gastric, and NSCLC patients (Table 2).

*(3) Nivolumab.* [BMS-936558, MDX-1106] A humanized IgG4 monoclonal antibody blocking PD-1. In phase I clinical trials, doses from 1 to 10 mg/kg every 2 weeks showed objective responses in 20–25% of patients with non-small-cell carcinoma (NSCLC), melanoma, and RCC [44]. Phase III trials are currently underway at 3 mg/kg dosing every 2 weeks, to evaluate its efficacy in renal cell carcinoma (RCC), NSCLC, and melanoma (Table 2).

*(4) Pembrolizumab.* Formerly known as Lambrolizumab [MK-3475], a humanized IgG4 monoclonal antibody binding to PD-1 it is the first anti-PD-1 agent approved by FDA. It is used in relapsed or refractory malignant melanoma following treatment with Ipilimumab or after treatment with Ipilimumab and a BRAF inhibitor in patients who carry a BRAF mutation at doses from 2 mg/kg to 10 mg/kg (Table 2).

*(5) MPDL3280.* It is a humanized IgG1 monoclonal antibody blocking PD-L1. In phase I setting, with doses ranging from 1 to 20 mg/kg every 3 weeks, an overall response (ORR) of 21% was seen in locally advanced or metastatic solid tumors such as melanoma, RCC, NSCLC, colon cancer, gastric cancer, head and neck squamous cell carcinoma (HNSCC), and lymphomas. Currently ongoing trials are evaluating its use in advanced melanoma, NSCLC, metastatic RCC, and metastatic urothelial bladder cancer (Table 2).

*(6) MS-936559.* A humanized PD-L1 Ig-G4 blocking monoclonal antibody [MDX-1105] blocks binding of PD-L1 to PD-1. Phase I studies in metastatic melanoma and NSCLC at doses ranging from 0.1 to 10 mg/kg every 2 weeks for up to 16 cycles, with 3 doses in each cycle being discontinued due to excellent results seen with Nivolumab.

*(7) Pidilizumab.* A humanized monoclonal IgG1 antibody [CT-011] blocks PD-1, the binding of PD-1 to PD-L1 and PD-L2. Early phases I-II trials are underway at doses of 0.2–0.6 mg/kg intravenously in diffuse large B-cell lymphoma (DLBCL) and metastatic colorectal cancer.

In animal studies, combination of immune check point inhibitors such as combining anti-CTLA4 and anti-PD1 antibodies enhanced effectory T-cell infiltration in tumor lesions resulting in decreased regulatory T-cell density [45]. There are clinical trials described below that use combination of immune check point inhibitors.

## 3. Malignancies Treated with Immune Checkpoint Blocking Monoclonal Antibodies

### 3.1. Malignant Melanoma

Stage IV melanoma treated with Dacarbazine for many years had dismal outcome with a median overall survival of 6–10 months and a 5-year survival rate of 10% [84, 85]. Immune therapy for melanoma focused on recombinant cytokines interferon alpha-2b (IFN *α*-2b) and interleukin-2 (IL-2). High dose IL-2 for advanced disease reported overall responses (ORR) of 5–27% and complete responses of up to 4% of patients [86]. High doses of IFN *α*-2b prolonged disease-free survival by 5% and, when used in the adjuvant setting, increased overall survival (OS) in high-risk patients by 3% [87].

Melanoma cells evade immune detection by downregulating surface HLA class I antigens with concomitant upregulation of nonclassical HLA-G antigen. Melanoma cells constitutively overexpress Fas receptor (FAS-R). FAS-ligand (FAS-L) is overexpressed on activated effector CD8+ T cells. Binding of FAS-R on the surface of melanoma cells to the FAS-L leads to apoptotic death of activated T-cells. Melanoma cells upregulate immune coinhibitory signals the PD-L1 (B7-H1) ligand and upon binding of these ligands on PD-1 receptor on T-cells immune suppressive cytokines are released from the malignant cell further impairing immune detection.

Ipilimumab, anti-CTLA4 human IgG1 antibody, showed sustained responses for longer than 2 years in metastatic malignant melanoma patients. A randomized, double-blind, dose-ranging clinical study with 88 patients of unresectable stage III or IV melanoma showed response rates as high as 11.1% and survival data of up to 30%. Grade 3 or 4 adverse events such as colitis, rash, and liver function abnormalities were observed in 19% of patients [39, 50, 88].

In combination with Dacarbazine, Ipilimumab increased response rates of 10% were seen with combination treatment when compared with single treatment controls [51, 52].

A phase III randomized trial showed that, at a dose level of 3 mg/kg, a median overall survival of 10 months was seen. When Ipilimumab was combined with an HLA-A^∗^0201-restricted gp100 vaccine peptide the median OS was 10.1 months; gp100 peptide treatment alone showed a median overall survival of 6.4 months [38]. Ipilimumab is FDA approved at dose of 3 mg/kg once every 3 weeks for 4 times for the treatment of unresectable or metastatic melanoma (stages III and IV disease). Of note, patients who experience immune-related adverse events are more likely to benefit from treatment with Ipilimumab.

Phase III study comparing Tremelimumab with chemotherapy (Dacarbazine and Temozolomide), indicated no increase in OS (12.6 m versus 10.7 m) and had similar ORR (10.7% versus 9.8%) except significantly longer response duration after Tremelimumab [54]. About 40% patients develop immune-related adverse events through the universal activation of T-cells, leading to tissue-specific inflammation and autoimmune related side effects, such as dermatitis, colitis and hepatitis, and panhypophysitis. The immune adverse events are best managed by systemic steroid treatment, without decrease in antibody therapy benefit [54].

Ten patients with metastatic melanoma treated with Nivolumab had benefit in a phase I study: one had partial response and one had tumor regression not meeting partial response (PR) criteria. Frequent side effects were decreased CD4+ count, lymphopenia, fatigue, and musculoskeletal events. Immune mediated events: inflammatory colitis, hypothyroidism, and polyarticular arthropathies were under 10% [55]. A phase I/II clinical trial with Nivolumab in 107 patients with stage IV melanoma showed sustained clinical response, which persisted even after cessation of therapy and a median OS of 16.8 months, 62% 1-year and 43% 2-year survival rates. Grades 3 and 4 toxicities were seen in only 5% of patients [56, 89].

Pembrolizumab (MK-3475, formerly called Lambrolizumab) was FDA approved in September 2014 for use in relapsed or refractory malignant melanoma following treatment with Ipilimumab or after treatment with Ipilimumab and a BRAF inhibitor in patients who carry a BRAF mutation. In early phase trial of a total of 135 patients with advanced melanoma, using doses from 2 mg/kg to 10 mg/kg response rates up to 38% were seen and a median progression-free survival >7 months [57]. There was a durable antitumor effect seen, with 87% of responders with ongoing response for more than 13 months of follow-up. At one year 81% of patients treated survived. Low grade fatigue, pruritus, and rash were common. If the tumors were PD-L1 positive an improved ORR and PFS by RECIST were seen (51% versus 6% in PD-L1 negative). One-year OS rate was 84% in PD-L1 positive and 69% in PD-L1 negative [58]. Baseline tumor size appears to be the strongest independent prognostic factor in metastatic melanoma patients treated with MK3475 [90]. Tumor size >90 mm was associated with a worse prognosis though these patients derived a benefit from MK-3475 achieving a median OS of 14 months.

A phase I trial Nivolumab (BMS-936559) of 207 advanced solid tumor patients included 55 patients with advanced melanoma; disease control was seen as early as 6 weeks in 46%. It induced durable tumor regression (ORR 6–17%) and prolonged stabilization of disease (12–41% at 24 weeks) was seen for all melanoma patients. Grade 3 or 4 toxic effects were seen in about 9% of patients [61].

A phase I clinical trial of Nivolumab (BMS-936559 IgG4 antibody to PD-1) in combination with Ipilimumab (IgG1 antibody to CTLA4) in 86 patients with stage III or IV melanoma (53 received concurrent therapy and 33 received sequential therapy) showed a 53% objective response rate at 2.5-year follow-up; in the concurrent treatment group there were marked tumor reductions of over 80% compared to 20% objective response rate (ORR) for sequenced treatments. While the combination therapy offers higher chances of response, increased ORR, and durable responses; toxicity with concurrent treatment was high with more than 50% of treated patients experiencing grades 3 and 4 toxicities [91]. Several phase III trials are currently underway to examine the safety and efficacy of combination regimens (Table 2).

### 3.2. Non-Small-Cell Lung Cancer

Metastatic non-small-cell lung cancer has dismal outcome with current treatments with only 4% of patients alive at 5 years despite intense multidisciplinary treatment. In metastatic lung cancer the tumor microenvironment favors the development of tolerant dendritic cells that drive the differentiation of T-cells towards immunosuppressive regulatory T-cells that release TGF-B [92]. TGF-B causes tolerance to tumor antigens. The presence of CD4+/CD8+ tumor-infiltrating lymphocytes in pathologic specimen has been associated with improved survival in [93] while it elevated levels of infiltrating immunosuppressive regulatory T-cells (CD4+ CD25+ FOXP3+) with increased risk of relapse [94].

A phase II randomized double-blind trial Ipilimumab in 204 stage IIIB and stage IV or recurrent NSCLC chemotherapy-naïve, randomized to carboplatin and paclitaxel, with placebo or Ipilimumab as either a concurrent or phased regimen was recently completed. The dose of Ipilimumab was 10 mg/kg in both arms. The concurrent arm used Ipilimumab with first 4 cycles and placebo with the last 2 cycles. The phased Ipilimumab arm assigned patients to placebo during the first 2 cycles of carboplatin and paclitaxel with Ipilimumab added with cycle #3 and continued for a total of 4 cycles [95]. Chemotherapy induces cancer cell death with release of tumor-associated antigens (TAA). TAA are able to amplify immune responses in the presence of immune check point inhibitors. Having more TAA to stimulate T-cells before Ipilimumab treatment seemed reasonable. The phased Ipilimumab had a significant increase in PFS: 5.1 months for phased regimen, 4.2 months for control regimen, and 4.1 months for concurrent regimen. Squamous histology was predictive of better outcomes in the phased arm. In currently ongoing phase III trial, NCT01285609 aims to validate these results and assess any change in OS.

In phase I clinical trial of 76 NSCLC patients treated with Nivolumab (BMS-936559 IgG4 antibody to PD-1) had response rates ranging from 6 to 32% when using doses of 1 mg/kg, 3 mg/kg, or 10 mg/kg every 2 weeks [63]. Thirty-three percent of patients with squamous cell histology and 12% of patients with nonsquamous cell histology showed an objective response, overall ORR 17%. PFS was 33% at 24 weeks with a median response duration of 74 weeks [64, 96]. More than half of the patients had a sustained response and had an OS of 42% at 1 year and 14% at 2 years. Among the 3 dose levels used, 3 mg/kg had highest objective response rate of 24%. Therapy was well tolerated, with only 9% grades 3-4 toxicities mainly fatigue, diarrhea, decreased appetite, nausea, and anemia.

In phase I clinical study of 38 patients with metastatic and locally advanced non-small-cell lung cancer who had previously been treated with two systemic chemotherapy regimens, Pembrolizumab (MK-3475) was given at 10 mg/kg every 3 weeks [66]. A 24% objective response rate was noted, including both squamous and nonsquamous subtypes and pretreatment PD-1 tumor expression was a predictor of response. Common adverse events were grades 1 and 2 fatigue, rash, pruritus, and diarrhea.

In a phase I clinical trial, 85 patients with heavily pretreated locally advanced and metastatic NSCLC patients were treated with MPDL-3280A, a humanized IgG1 monoclonal antibody against PD-L1. Twenty-four percent of patients had objective response in squamous and nonsquamous cell histologies and at 24 weeks PFS rate was 46% [67]. One hundred percent of patients with PDL-1 positive tumors had treatment response. Response rates were different according to smoking status, the former or current smokers had 25% versus 16% in never-smoker, indicating that immune stimulation may detect cancer cells after carcinogen exposure.

### 3.3. Renal Cell Carcinoma

A phase I clinical trial of 34 patients with metastatic renal cell carcinoma Nivolumab (BMS-936559 IgG4 antibody to PD-1) at doses of 1 mg/kg and 10 mg/kg achieved objective responses in 9 patients and stable disease lasting >24 weeks in another 9 patients [63].

A double-blinded phase II randomized trial of Nivolumab (BMS-936559 IgG4 antibody to PD-1) dose-ranging monotherapy, at 0.3, 2, or 10 mg/kg intravenously every 3 weeks until progression or toxicity in 168 heavily pretreated (four or less lines of therapy) metastatic clear-cell RCC patients showed an objective response in 20 to 22% of patients and median OS of 18.2 months in the lowest dose group [44]. The responses were durable, lasting longer than 12–20 months. Nivolumab was overall well tolerated, with grades 3-4 CTCAE adverse events in less than 17% patients. The median PFS ranged from 2.7 to 4.2 months. No clear dose-response relationship was seen, suggesting that even low doses of anti-PD1 may elicit significant clinical benefit likely due to binding affinity of these antibodies to their targets.

Phase I clinical trials of combinations Nivolumab (BMS-936559 IgG4 antibody to PD-1) with the VEGF receptor TKIs, Pazopanib, and Sunitinib were based on the rationale that PD-1 and VEGF inhibition may have additive benefit for intratumoral immune environment by decreasing immune suppressive cell populations and suppressing effects of VEGF on dendritic cell function [74]. Among the 33 patients in the Nivolumab (2 mg/kg every 3 weeks and 5 mg/kg every 3 weeks) and Sunitinib arm (at dose 50 mg/kg, 4 weeks on and 2 weeks off), objective response was seen in 52% patients, most as early as 6 weeks. In this arm, the PFS rate was 78% at 24 weeks and the median PFS was 12 months, but this included mostly treatment of naïve patients. The combination of Sunitinib and Nivolumab slightly increased OR (52%) and PFS (12 months) compared to Sunitinib alone arm OR (47%) and PFS (9.5 to 11 months). This minimal survival advantage at a cost of significant toxicity over monotherapy will remain a challenge in bringing such combination therapies to clinical practice (Table 2).

In metastatic RCC at two different dose combinations of Nivolumab in combination with the anti-CTLA-4 antibody Ipilimumab was studied in a phase I/II clinical trial: Nivolumab dose of 3 mg/kg and Ipilimumab dose of 1 mg/kg versus Nivolumab dose of 1 mg/kg and Ipilimumab dose of 3 mg/kg in 44 patients with metastatic renal cell carcinoma [76]. The overall response rate was 39% and PFS ranging from 9 to 10 months with prolonged antitumor effects. CTCAE grades 3-4 gastrointestinal and liver toxicities were seen in 43% patients. Phase III clinical trials of Nivolumab in renal cell carcinoma and combination studies are ongoing.

In a phase I clinical trial of MPDL3280A (humanized IgG1 monoclonal antibody against PD-L1), 53 patients with metastatic renal cell carcinoma (RCC) were enrolled. More than 80% patients had prior systemic therapy; MPDL3280A was administered intravenous every 3 weeks at doses between 3 and 20 mg/kg [75]. CTCAE grades 3-4 adverse events were seen in 13% of patients. Responses per RECIST criteria were seen across all doses with prolonged interval of stable disease and at 24 month PFS was 50% [75].

A phase I/II trial of Pembrolizumab (MK-3475, formerly called Lambrolizumab) in combination with Pazopanib in advanced clear cell RCC patients is currently underway to assess safety, efficacy, and response. This trial analysis of patients with RCC treated with Pazopanib showed that patients' whose tumors have high expression of PD-L1 have shorter PFS (NCT02014636).

### 3.4. Bladder Cancer

Phase I trial of MPDL3280A (humanized IgG1 monoclonal antibody blocking PD-L1) in 31 patients with heavily pretreated metastatic urothelial bladder cancer showed a 50% ORR [97]. The treatment was well tolerated and the responses were rapid and durable with median OS of 6-7 months. Responses were reported according to expression of PD-L1 by IHC on tumors. PD-L1 positive tumors, defined as immunohistochemistry score (IHC) of 2 or 3, had a 43% response rate by RECIST criteria, while tumors with (IHC score of 0 or 1) had 11% response rate. The study showed that expression of PD-L1 status changes over time and should not be used as a reliable marker for clinical applications. CTCAE adverse events grades 3-4 were seen in 3.2% of patients which showed a 50% ORR [97].

### 3.5. Prostate Cancer

A phase I/II trial in 33 men with metastatic castrate-resistant prostate cancer (mCRPC) studied Ipilimumab alone or in combination with radiotherapy, given 24–48 h prior to Ipilimumab, either before or after chemotherapy [71]. At the highest dose level (10 mg/kg), immune-related adverse events (irAEs) were manageable and included colitis (16%), diarrhea (8%), and hepatitis (10%). Of the 50 patients in the 10 mg/kg dose level cohort, 8 had a ≥50% decline in PSA. Of the 28 subjects in the 10 mg/kg cohort with evaluable tumors, one had a complete response and six had stable disease [71]. CTCAE adverse events were GI and skin related, with only 10% having grades 3-4 [71, 72]. Two phase III trials evaluating Ipilimumab in men with metastatic castrate-resistant prostate cancer are in progress.

A phase III trial conducted in this setting did not show any improved overall survival in the Ipilimumab treatment group but was positive for one of its secondary endpoints time to progression [72]. The subgroup analysis suggested that Ipilimumab was beneficial in mCRPC patients with no visceral metastases and a favorable performance status.

In phase I/II trials, the combinations were generally well tolerated and had an acceptable toxicity profile [98–101]. In spite of preclinical rationale for dual blockade of PD-1/PD-L1 in prostate cancer, no objective responses were seen in the prostate cancer cohorts in phase I PD-1 and PD-L1 trials [98–101] (Table 2).

Combination immunotherapy trials of Ipilimumab with GM-CSF or GVAX have been conducted. In phase I/II trials, the combinations were generally well tolerated and had an acceptable toxicity profile [98–101] (Table 2).

### 3.6. Ovarian Cancer

A phase I/II trial of 11 patients with FIGO stage IV ovarian cancers previously treated with either chemotherapy or GVAX (a vaccine product comprised of autologous, irradiated tumor cells engineered to secrete the immune stimulatory cytokine, granulocyte macrophage colony-stimulating factor) used Ipilimumab at a dose level of 3 mg/kg. Two patients had CTCAE grade 3 gastrointestinal inflammatory adverse events [77, 78]. Two patients had exceptional responses: one patient a dramatic fall of serum CA125 levels and a substantial regression of a large hepatic metastasis, mesenteric lymph nodes, and an omental caking; another patient had reduction in pain and ascites and stabilization of CA125 levels [78]. Four other patients had stable disease as assessed by blood CA125 levels and imaging that lasted longer than 10 months [77, 78].

### 3.7. Squamous Cell Carcinoma of Head and Neck

Recurrent squamous cell carcinoma of head and neck (SCCHN) is incurable with palliative chemotherapy resulting in a median survival of 8–10 months. SCCHN tumors express high levels of PD-L1 expression in 46–100% in the primary, recurrent, and metastatic settings [83, 102–106].

Human papilloma virus (HPV) has prognostic significance in oropharyngeal SCCHN and there is higher expression of PD-L1 in the HPV positive patients [32, 83, 103]. HPV infection of oropharyngeal epithelium is accompanied by PD-L1 expression creating a pseudo-immuno-privileged site for the developing malignancy. While PD-1 expression on effector T-cells is seen in both HPV positive and negative SCCHN tumors, the degree of expression seems to be increased in those patients with HPV positive disease [32, 83]. Regulatory T-cells which mediate peripheral tolerance, suppress effector T-cells, and inhibit immune mediated destruction are increased in the blood and tumor microenvironment in patients with SCCHN [107]. In SCCHN, a higher frequency of intratumoral regulatory T-cells expressing PD-1 and CTLA-4 have higher suppressive effects on immunity when compared to peripheral blood regulatory T-cells [108]. These data suggest that blocking the interaction between PD-L1 and PD-1 may trigger cellular antitumor immune response, in recurrent and metastatic SCCHN patients. Two ongoing phase I trials, with anti-PD-L1mAb MEDI4736 (IgG1 isotype) (NCT01693562) and MK-3475 anti-PD-1 monoclonal Ab (IgG4 isotype) (NCT01848834), include cohorts of recurrent/metastatic SCCHN patients.

## 4. Discussion

Cancer immunotherapy showed promising results in several solid tumors. The benefits of such therapies in metastatic incurable solid tumors exceed those seen with conventional cytotoxic chemotherapy. Harnessing the immune system to eliminate tumors has limitations most of them being off-target autoimmune side effects. Immunotherapy has the potential for durable response and significantly improved long-term survival, potentially even on treatment breaks. There are no biomarkers to predicting exceptional responders or the patients at risk for immune-related toxicity. Choueiri et al in a prospective randomized clinical trial of Nivolumab in previously treated and untreated metastatic RCC showed that responses correlated to expression of PD-L1 by immunohistochemistry (IHC) in tumors [73]. Responses were higher in tumors staining positive (IHC 2 or 3) for PD-L1 of 22% versus 8% in IHC 0-1 tumors [73]. Changing in tumor heterogeneity in time secondary to mutation accumulation and tumor immune editing will result in changes in PD-L1 expression over time. Additionally CD3 and CD8 T-cell infiltration in tumor environment appeared to correlate with clinical responses. Prognostic implications of PD-1 and PD-L1 in staining in SCCHN combined with data from phase 1 anti-PD-1 and anti-PD-L1 trials supports that immune staining of tumors for PD-1 and PD-L1 will help guide immune therapy [63, 109, 110].

PD-L1 and PD-L2 status by immunohistochemistry was independent predictor of prognostic factor in postoperative esophageal cancer patients [22]. Of the 41 patients evaluated, 18 were positive for PD-L1 or PD-L2 expression and 23 were negative. PD-L positive patients had a significantly poorer prognosis with worse OS than the negative patients. The OS was worse with tumor positive for both PD-L1 and PD-L2, than those with tumor negative for both 50% versus 100% 1-year survival [22]. Significant differences were also noted in 1-year survival rate after surgery between positive and negative patients of PD-L1 and PD-L2 with T2, T3 disease, and stage III cancer [22]. The effect of PD-L status on postoperative prognosis was more pronounced in the advanced stage of tumor than in the early stage. PD-L2 expression was inversely correlated with tumor-infiltrating CD8+ T-cells but not PD-L1 expression [22].

## 5. Conclusion

Therapies that limit immune suppression in malignancy are effective in prolonging survival in cancer patients. The effect of immune check point inhibitors appears limited in patients with advanced bulky tumors likely due to highly suppressive effect of the tumor microenvironment and established tolerance mechanisms. As a consequence, immune based therapies may be more effective in patients with low volume disease such as earlier stage cancers immediately after bulk cytoreduction or chemotherapy use and may be more effective in treatment of minimal residual disease. Tumors characterized by epithelial-mesenchymal transition [EMT] have high expression for immune inhibitory molecules like PD-1, PD-L1, PD-L2, and CTLA-4 and may be more amenable to treatment with immune of checkpoint inhibitors [111]. A major limitation of immune checkpoint inhibitors is lack of information on binding affinities to antigens on human immune cells and cancer cells to control for the unforeseen variation in protein function from individual to individual. Such variation in protein function amongst individuals may explain the off-target immune and adverse effects seen.

## Figures and Tables

**Figure 1:**
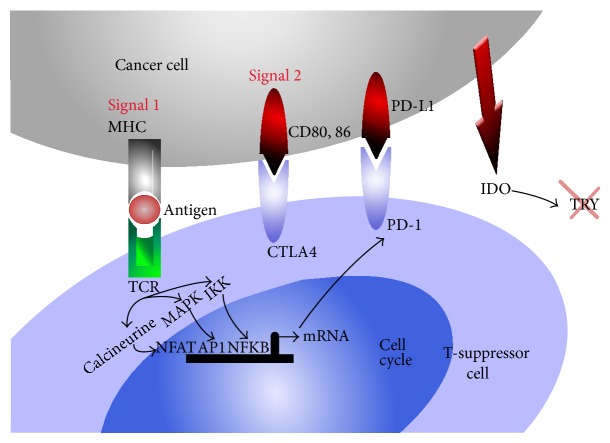
Cancer cell mediated immune suppression: upon ligation CTLA4 or PD-1 on suppressor cells, cancer cells produce 2,3 indolamine dioxygenase (IDO) and others (arginase, nitric oxide synthase) degrading amino acids arginine and tryptophan necessary for immune detection and elimination function of the effector CD8+ T-cells. Cancer cells undergo phenotypic or genomic modification under immune attack resulting in the survival and selection of variants that are capable of escaping immune attack. These modifications include HLA class 1, loss of tumor antigens, lack of death receptor signaling, regulatory T-cells, inhibitory cytokines, and immune check point molecules. Ligation of PD-L1 on the tumor cell surface results in tumor protection from cell death [82]. Interactions between PD-L1 and PD-1 in the tumor microenvironment protect the tumor through several distinct pathways including the ligation of PD-1 by PD-L1 on antigen specific T-cells leading to functional anergy and/or apoptosis of these effector T-cells [19, 83]. Effector T cells are further inhibited by PD-L1:CD80 interactions [18]. While PD-L1 interactions with PD-1 on cytotoxic CD8 T-cells dampen tumor specific effector immunity, PD-L1 PD1 interaction on T-regulatory cells (CD4+, CD25+) increases their suppressive function [35–37]. IDO: indolamine 2,3 deoxygenase; TCR: T cell receptor; MHC: major histocompatibility antigen; IL-2: interleukin-2; CTLA4 cytotoxic T lymphocytes antigen-4; PD-1: programmed death-1 also known as CD279; PD-L1 programmed death-ligand 1 known as B7-H1 or CD274; PD-L2 programmed death-ligand 2, known as CD273 or B7-DC B-7 dendritic cell; IFN-*γ*: interferon gamma; IL-2: interleukin-2; APC: antigen presenting cells; TRY: tryptophan, AP-1: activator protein-1; NFAT: nuclear factor of activated T-cells; NFKB: nuclear factor kappa B; MAPK: mitogen activated protein kinase; IKK: I kappa B kinase; PI3K: phosphatidylinositol-4,5-biphosphate 3-kinase; CDK: cyclin dependant kinase; JAK3: Janus kinase 3; mTOR: mammalian target of rapamycin.

**Table 1:** B-7 family of receptor ligands expressed by antigen presenting cells and various malignancies: CD cluster derivation; B7.1 known as CD80; B7.2 known as CD86; CD-28 and CD-152 present on T-naïve cells; ICOS inducible costimulatory ligand; (+) with and (−) without; CD152 also known as CTLA4 cytotoxic T lymphocytes antigen-4; PD-1 programmed death-1 also known as CD279; PD-L1 programmed death-ligand 1 known as B7-H1 or CD274; PD-L2 programmed death-ligand 2, known as CD273 or B7-DC B-7 dendritic cell; IFN-*γ* interferon gamma; IL-2 interleukin 2; Ref reference.

B-7 family molecules	CD-designation	Major ligands on immune cells	Role immunity activation versus immune suppression	Malignancies expressing B-7 molecules	Ref
B7.1	CD80	CD28; CTLA4 (CD152)	(+) 2nd signal activation (−) second signal anergy	CD-80 on acute myeloid leukemia cells	[37]

B7.2	CD86	CD28; CTLA4 (CD152)	(+) 2nd signal activation (−) second signal anergy	CD-86 on chronic lymphatic leukemia cells	[46]

B7-H1 (PD-L1)	CD274	PD-1 (CD279)	Ligating PD-1 on T-cells suppresses CD8+ response	None	[47]

B7-H2; B7-H3; B7-H6; ICOS	CD275	CD278	Immune suppression	Dendritic cells infiltrating malignancies; cancer cells: hematologic malignancies, breast, gastrointestinal, lung, melanoma, bladder, and genitourinary cancers	[48]

PD-L2 B7-DC	CD273	PD-1 (CD279)	Immune suppression reduces IL-2 and IFN-*γ* secretion, decreases proliferation and cytotoxicity, and induces apoptosis in activated T-cells	Primary mediastinal (thymic) large B-cell lymphoma	[49]

**Table 2:** Currently available immune check point inhibitors in clinical use. CD152 also known as CTLA4 cytotoxic T lymphocytes antigen-4; PD-1: programmed death-1 also known as CD279; PD-L1: programmed death-ligand 1 known as B7-H1 or CD274; PD-L2: programmed death-ligand 2, known as CD273 or B7-DC B-7 dendritic cell; IFN-*γ*: interferon gamma; IL-2: interleukin-2; AE: adverse events; NSCLC: non-small-cell carcinoma; RCC: renal cell carcinoma, ORR: overall response; HNSCC: head and neck squamous cell carcinoma; OS: overall survival; PFS: progression-free survival; RECIST: response evaluation criteria in solid tumors; vs: versus; gp100: vaccine glycoprotein 100 vaccine; FIGO: Federation International for Gynecologic Oncology; RCC: renal cell carcinoma; CRPC: castrate resistant prostate cancer; SCC: squamous cell carcinoma.

Type of cancer	Drug	Target	Clinical trial, Phase	Outcome	Ref
Malignant melanoma	Ipilimumab (FDA approved in 2011)	CTLA-4	NCT00094653, III	676 unresectable stage III or IV melanoma treated with Ipilimumab vs gp100 vaccine. Improved survival with Ipilimumab, with median OS 10 months. Grades 3-4 AEs seen only in 10–15% patients.	[38]
	Ipilimumab	CTLA-4	I/II	Among 88 patients with unresectable stage III/IV melanoma treated with Ipilimumab at different dose levels, 7 had stable disease and 2 had response. Grades 3-4 AEs were only seen in 14% patients.	[50]
	Ipilimumab + Dacarbazine vs Dacarbazine	CTLA-4	NCT00324155, III	502 patients with untreated metastatic melanoma were given Ipilimumab + Dacarbazine vs Dacarbazine alone. Median OS was 11 months in combination arm vs 9 months with Dacarbazine only arm.	[51]
	Ipilimumab + Dacarbazine vs Ipilimumab	CTLA-4	NCT00050102, II	72 patients with unresectable, metastatic melanoma received Ipilimumab with Dacarbazine or Ipilimumab alone. ORR was 14.3% with median OS 14.3 months in first arm vs ORR of 5.4% with median OS 11.4 months in second arm.	[52]
	Tremelimumab	CTLA-4	NCT00086489, I/II	28 patients with metastatic melanoma received escalating (3, 6, and 10 mg/kg) doses of Tremelimumab. Durable antitumor responses were seen and drug was well tolerable.	[53]
	Tremelimumab	CTLA-4	NCT00257205, III	In 534 patients with treatment naïve, unresectable stage III or IV melanoma, median OS was 12.6 months in Tremelimumab arm vs 10.7 months in Temozolamide or Dacarbazine arm. ORR was similar in both arms but response duration was 35.8 months vs 13.7 months. No significant survival advantage was seen.	[54]
	Nivolumab	PD-1	NCT00441337, I	10 patients with advanced metastatic melanoma showed evidence of antitumor activity with Nivolumab and drug was well tolerated.	[55]
	Nivolumab	PD-1	NCT00730639, I	Among 107 advanced melanoma patients treated with Nivolumab, 33 had objective tumor regression, with a response duration of 2 years, and median OS was 16.8 months.	[56]
	Pembrolizumab (MK-3475)	PD-1	NCT01295827, I	MK-3475 was used in 173 patients with advanced melanoma who progressed after Ipilimumab. ORR was 26% and treatment was well tolerated.	[57–59]
	MPDL3280A	PD-L1	NCT01375842, I	In 45 melanoma patients, MPDL3280A was well tolerated and an ORR of 26% was observed. 24-week PFS was 25%.	[60]
	BMS-936559	PD-L1	NCT00729664, I	Among 55 melanoma patients, durable tumor responses and prolonged stable disease were seen.	[61]

Lung	Ipilimumab	CTLA-4	II	An objective response rate of 19% for NSCLC patients with squamous histology and 15% with nonsquamous histology. Patients who received phased Ipilimumab and Carboplatin and Paclitaxel showed improved PFS as compared to Carboplatin/Paclitaxel alone.	[62]
	Nivolumab	PD-1	I	Among 6 heavily pretreated patients with NSCLC, one had partial remission for over 14 months and other 5 had stable disease.	[55]
	Nivolumab	PD-1	I	Among 129 NSCLC patients, 17% had objective responses. Best response of 24% was seen at 3 mg/kg dose and median OS was 14.9 months. Durable and rapid responses, with OS 42% at 1 year and 24% at 2 years, across all histological subtypes. Median OS was 9.2 m in squamous and 10.1 m in nonsquamous.	[63–65]
	Pembrolizumab (MK-3475)	PD-1	I	Among 38 patients with advanced NSCLC who received >2 prior therapies, responses as early as 9 weeks were seen in 24% patients, both in squamous and non-squamous histologies. Median OS was 51 weeks.	[66]
	BMS-936559	PD-L1	I	Among 49 patients with advanced NSCLC, objective responses were seen in 6 patients and another 6 with stable disease (both squamous and nonsquamous subtypes).	[61]
	MPDL-3280A	PD-L1	NCT01375842, I	85 patients with NSCLC were evaluated for safety and 53 for efficacy. ORR was 21% with higher rates in PD-L1 positive tumors. Responses were sustained and dramatic response was seen in the smoking cohort.	[67, 68]
	MEDI-4736	PD-L1	NCT01693562, I	In 13 heavily retreated NSCLC patients (median 4 lines of prior treatment), 3 patients achieved PR and 2 with response not reaching PR as early as 6 weeks. Response was durable. Acceptable safety profile at all doses.	[69]
	Tremelimumab vs best supportive care	CTLA-4	NCT00312975, II	Among 87 patients with locally advanced or metastatic NSCLC, no superiority in PFS was seen in study arm over BSC. 4.8% ORR was seen.	[70]

Metastatic CRPC	Ipilimumab	CTLA-4	NCT00323882, I/II	50 CRPC patients received Ipilimumab 10 mg/kg and RT and had manageable AEs. Eight patients had PSA decline >50%, 1 had CR and 6 had stable disease.	[71]
	Ipilimumab	CTLA-4	III	No difference in OS with Ipilimumab vs placebo in post-Docetaxel CRPC and bone metastasis following radiation therapy. PFS advantage was seen with Ipilimumab.	[72]
	Nivolumab (BMS-936558)	PD-1	NCT00730639, I	No objective responses were seen in 17 CRPC enrolled.	[63]

Metastatic RCC	Nivolumab	PD-1	NCT01354431, II	168 patients with metastatic clear cell RCC, median duration of response was 15.7 months and median OS was 18.2 months. 54% of responses lasted >12–20+ months.	[44]
	Nivolumab	PD-1	NCT01358721, I	91 patients with metastatic RCC, Nivolumab showed clinical activity in previously treated and untreated metastatic RCC [ORR 16%]. Median duration of response was 15 months. Responses were higher in PD-L1+ patients (ORR 22%) but also seen in PD-L1 patients (ORR 8%).	[73]
	Nivolumab + Sunitinib or Pazopanib	PD-1	NCT01472081, I	Seven patients with metastatic RCC received Nivolumab in combination with Sunitinib (S) (33 patients) or Pazopanib (P) (20 patients). 41% had responses as early as 6 weeks, with ORR 52% in (S) arm; 56% has responses as early as 6 weeks, with ORR 45% in (P) arm PFS at 24 weeks was 78% for S arm and 55% for (P) arm.	[74]
	MPDL3280A	PD-L1	NCT01375842, I	53 patients with metastatic RCC were evaluated for efficacy and safety. RECIST responses were observed across all doses and some has prolonged stable disease prior to RECIST response. 24-month PFS was 50%.	[75]
	Nivolumab + Ipilimumab	CTLA-4	NCT01472081, I	ORR was 29% in Nivolumab (N) and Ipilimumab (I) (N-3 mg/kg and I-1 mg/kg) arm. ORR was 39% in N-1 mg/kg and I-3 mg/kg arm. Stable disease was seen in 33% in N3 + I1 arm and 39% in N1 + I3 arm.	[76]

Urothelial bladder	MPDL3280A	PD-L1	NCT01375842, I	ORR was 50% with a median time to response of 43 days, among 31 metastatic urothelial bladder cancer patients, including visceral metastases.	[71]

Ovarian	Ipilimumab	CTLA-4	I	Among 2 pretreated advanced ovarian cancer patients, CA-125 level stabilization was seen in one and reduction in the other.	[77]
	Ipilimumab	CTLA-4	I	Among 11 patients with FIGO stage IV ovarian cancer, who previously received GVAX-antitumor activity was seen in one with dramatic fall in CA-125 and regression of metastatic lesions. Another 5 patients had stable disease per CA-125 and imaging.	[78]
	MDX-1105 (BMS-936559)	PD-L1	NCT00729664, I	Among 17 patients with ovarian cancer, 1 has partial response and 3 had stable disease lasting at least 24 weeks, all at 10 mg/kg dose.	[61]

SCC head and neck	MPDL3280A	PD-L1	NCT01375842, I/II	One patient with metastatic head and neck cancer had response by second cycle of therapy.	[79]
	MK3475	PD-1	NCT01848834, IB	On interim analysis of 60 patients with metastatic or recurrent head and neck cancer, drug was well tolerated. Tumor shrinkage was seen in many patients, but protocol specific analysis is pending.	[80]
	MEDI4736	PD-L1	NCT01693562, I	Preliminary data suggests that even in heavily pretreated patients of head and neck cancer, tumor shrinkage was detectable as early as 6 weeks.	[81]
